# All-Cause Mortality in People With Four-Class Drug-Resistant HIV: A Matched Cohort Analysis With Data From the PRESTIGIO Registry

**DOI:** 10.1093/cid/ciaf421

**Published:** 2025-08-01

**Authors:** Andrea Giacomelli, Nicolò Capra, Riccardo Lolatto, Roberta Gagliardini, Tommaso Clemente, Leonardo Calza, Carlo Torti, Filippo Lagi, Chiara Fornabaio, Giulia Marchetti, Giancarlo Orofino, Vincenzo Spagnuolo, for PRESTIGIO Study Group, Antonella Castagna, Vincenzo Spagnuolo, Daniele Armenia, Stefano Bonora, Leonardo Calza, Anna Maria Cattelan, Giovanni Cenderello, Adriana Cervo, Laura Comi, Antonio Di Biagio, Emanuele Focà, Roberta Gagliardini, Andrea Giacomelli, Filippo Lagi, Giulia Marchetti, Stefano Rusconi, Francesco Saladini, Maria Mercedes Santoro, Maurizio Zazzi, Andrea Galli, Daniele Armenia, Francesco Saladini, Maria Mercedes Santoro, Maurizio Zazzi, Elisabetta Carini, Sabrina Bagaglio, Girolamo Piromalli, Riccardo Lolatto, Marcello Tavio, Alessandra Mataloni Paggi, Ornella Schioppa, Valentina Da Ros, Annalisa Saracino, Flavia Balena, Laura Comi, Daniela Valenti, Claudia Suardi, Pierluigi Viale, Leonardo Calza, Federica Malerba, Silvia Cretella, Riccardo Riccardi, Francesco Castelli, Emanuele Focà, Davide Minisci, Francesca Pennati, Barbara Menzaghi, Maddalena Farinazzo, Bruno Cacopardo, Maurizio Celesia, Michele Salvatore Paternò Raddusa, Carmen Giarratana, Paolo Fusco, Vincenzo Olivadese, Angelo Pan, Chiara Fornabaio, Paola Brambilla, Alessandro Bartoloni, Filippo Lagi, Paola Corsi, Seble Tekle Kiros, Filippo Ducci, Susanna Giachè, Cecilia Costa, Alessio Bellucci, Elisa Mirabelli, Teresa Santantonio, Sergio Lo Caputo, Sergio Ferrara, Arianna Narducci, Emanuele Pontali, Marcello Feasi, Antonio Sarà, Matteo Bassetti, Antonio Di Biagio, Sabrina Blanchi, Antonella Castagna, Vincenzo Spagnuolo, Camilla Muccini, Elisabetta Carini, Sabrina Bagaglio, Riccardo Lolatto, Andrea Galli, Rebecka Papaioannu Borjesson, Tommaso Clemente, Girolamo Piromalli, Spinello Antinori, Andrea Giacomelli, Tiziana Formenti, Fabiola Schiavo, Giulia Marchetti, Lidia Gazzola, Fabiana Trionfo Fineo, Massimo Puoti, Cristina Moioli, Federico D’Amico, Cristina Mussini, Adriana Cervo, Elio Manzillo, Amedeo Lanzardo, Anna Maria Cattelan, Maria Mazzitelli, Antonio Cascio, Marcello Trizzino, Elisa Fronti, Diletta Laccabue, Federica Carli, Roberto Gulminetti, Layla Pagnucco, Mattia Demitri, Daniela Francisci, Giuseppe De Socio, Elisabetta Schiaroli, Elisa Garlassi, Romina Corsini, Roberta Gagliardini, Marisa Fusto, Loredana Sarmati, Vincenzo Malagnino, Tiziana Mulas, Mirko Compagno Carlo Torti, Simona Di Giambenedetto, Silvia Lamonica, Pierluigi Francesco Salvo, Giovanni Cenderello, Rachele Pincino, Mario Tumbarello, Massimiliano Fabbiani, Francesca Panza, Ilaria Rancan, Giovanni Di Perri, Stefano Bonora, Micol Ferrara, Andrea Calcagno, Silvia Fantino, Stefano Nardi, Marta Fiscon

**Affiliations:** Department of Biomedical and Clinical Sciences, Università Degli Studi di Milano, Milan, Italy; III Infectious Diseases Unit, ASST Fatebenefratelli Sacco, Luigi Sacco Hospital, Milan, Italy; IRCCS Ospedale San Raffaele, Infectious Diseases Unit, Milan, Italy; IRCCS Ospedale San Raffaele, Infectious Diseases Unit, Milan, Italy; Immunodeficiency Unit, National Institute for Infectious Diseases Lazzaro Spallanzani, IRCCS, Rome, Italy; IRCCS Ospedale San Raffaele, Infectious Diseases Unit, Milan, Italy; Department of Medical and Surgical Sciences, Unit of Infectious Diseases, S. Orsola Hospital, IRCCS Azienda Ospedaliero-Universitaria di Bologna, Bologna, Italy; Infectious Diseases Unit, Agostino Gemelli; University Hospital, Rome, Italy; Infectious and Tropical Diseases Unit, Careggi University Hospital, Florence, Italy; Infectious Diseases Unit, ASST Cremona, Cremona, Italy; Clinic of Infectious Diseases and Tropical Medicine, San Paolo Hospital, ASST Santi Paolo e Carlo, Department of Health Sciences, University of Milan, Milan, Italy; Division I of Infectious and Tropical Diseases, ASL Città di Torino, Torino, Italy; IRCCS Ospedale San Raffaele, Infectious Diseases Unit, Milan, Italy; School of Medicine, Vita-Salute San Raffaele University, Milan, Italy

**Keywords:** death, immune suppression, multidrug resistance, outcome, heavily treatment experienced

## Abstract

People with human immunodeficiency virus (HIV) (PWH) with 4-drug class resistance (4DR) had a higher risk of death than non-4DR PWH, primarily due to lower CD4 cell counts. The priority for this vulnerable population is achieving virological control to enable immune recovery.

Heavily treatment-experienced (HTE) people with human immunodeficiency virus (HIV) (PWH) are at increased risk of adverse clinical outcomes, including malignancies and major cardiovascular events, compared with PWH without extensive drug resistance [1–3]. This elevated risk is primarily attributable to chronic inflammation, driven by prolonged periods of unsuppressed viremia, and exposure to complex antiretroviral regimens (ART) with significant metabolic toxicities [4–6]. Previous studies have highlighted poor prognosis among individuals with resistance to 3 drug classes [7, 8]. However, it remains unclear whether PWH with 4-drug-class resistance (4DR-PWH), a subset of HTE with extremely limited therapeutic options, have a higher risk of mortality compared with PWH without multidrug-resistant viruses [1]. This study aims to evaluate whether individuals with 4DR-PWH have an elevated risk of death compared with those without 4DR (non-4DR-PWH).

## METHODS

This retrospective, propensity score-matched cohort study investigated adult PWH under ART. 4DR-PWH (exposed group) were compared with non-4DR-PWH (unexposed group). 4DR-PWH were identified from the PRESTIGIO Registry (NCT04098315) and matched to ≥1 non-4DR-PWH from the Centro San Luigi (CSL) HIV Cohort. The PRESTIGIO Registry is a multicenter, Italian, registry-based cohort of adults with HIV resistant to NRTIs, NNRTIs, PIs, and INSTIs (in the absence of INSTI genotypic resistance data, resistance was approximated as virological failure with ≥2 consecutive HIV-RNA >50 copies/mL). Its protocol was approved by the Ethics Committees of participating centers [9]. Non-4DR-PWH were obtained from the CSL cohort, which includes PWH followed at the IRCCS San Raffaele Scientific Institute, Milan, Italy (Ethics Committee approval no. 34/int/December 2017) [2]. Non-4DR-PWH should have no history of resistance to more than 2 drug classes. They were matched to 4DR-PWH at a ratio of 4:1 based on age, sex assigned at birth, and ART duration. An index date (baseline) was assigned to each exposed and unexposed individual: for exposed, this was the date of first evidence of 4DR; for unexposed, this was the index date of the corresponding case. Sex assigned at birth was treated as an exact matching criterion. A nearest-neighbor method with a caliper distance of ±3 years was employed to match for age and ART duration. Propensity scores were calculated using a logistic regression model. Overall, 212 exposed were matched with 4 unexposed, 2 with 3 unexposed, 1 with 2 unexposed, and 13 with one unexposed.

The primary outcome of the study was death (ascertained with regional and national health care registries). Causes of death were also collected and classified into 6 categories: AIDS-related, liver-related, non-AIDS cancer, other non-AIDS conditions, suicide, and unknown causes.

Baseline characteristics of the study population were summarized as medians (interquartile ranges [IQRs]) for continuous variables and frequencies (percentages) for categorical variables. Comparisons were performed using χ^2^ or Fisher's exact tests for categorical data and the Mann–Whitney U test for continuous data, as appropriate. Incidence rates (IRs) of death, along with 95% confidence intervals (CIs), were calculated using a univariable Poisson regression model and expressed per 100 person-years of follow-up (PY). Incidence rate ratios (IRRs) with 95% CIs were estimated to compare the exposed (4DR) and unexposed (non-4DR) groups. Follow-up time was defined from baseline to the last available visit (censoring date: 12 April 2024). Cumulative probabilities of death and corresponding 95% CIs were estimated using Kaplan–Meier curves and compared with the log-rank test. Univariable and multivariable Cox proportional hazards models were used to assess the effect of 4DR status on mortality. The multivariable model adjusted for selected comorbidities occurred before baseline (hypertension, diabetes, major cardiovascular events, chronic kidney disease, hepatitis C, and cancer). In alternative models, we also adjusted (1) for previous AIDS defining events and (2) for baseline CD4 cell count. We estimated natural direct and indirect effects of 4DR on all-cause mortality via mediation analysis using marginal structural models (MSM) [10], with CD4 as mediator and clinical comorbidities as confounders. CD4 was Box–Cox transformed and modeled via linear regression to estimate MSM weights. A two-sided *P*-value <.05 was considered statistically significant. All statistical analyses were performed using R software (R Foundation for Statistical Computing, Vienna, Austria, version 4.3.1).

## RESULTS

A total of 228 4DR-PWH and 869 non-4DR-PWH were included, contributing 2003 and 7259 PY of follow-up, respectively, with a median overall follow-up of 8.67 years (IQR 5.59–11.29). Participants’ characteristics are detailed in Table 1. During the follow-up period, 28/228 (12.3%) 4DR-PWH and 67/869 (7.7%) non-4DR-PWH died, corresponding to IRs of 1.4/100 PY (95% CI .93–2.02), and .92/100 PY (95% CI .72–1.17), respectively [IRR of 1.51 (95% CI .97–2.35, *P* = .063)]. Causes of death in PWH with and without 4DR are reported in Supplementary Table 1. The 5- and 10-year probability of death in 4DR-PWH compared with non-4DR-PWH were 4.62% (95% CI 1.78–7.38) versus 4.14% (95% CI 2.75%–5.52%) and 12.37% (95% CI 7.28%–17.58%) versus 8.76% (95% CI 6.42%–11.05%), respectively (Supplementary Figure 1). In univariable analysis, 4DR-PWH had a nonsignificantly higher risk of death compared with non-4DR-PWH [hazard ratio (HR) 1.50, 95% CI .97–2.34, *P* = .07]. Multivariable analysis adjusted for comorbidities showed a significantly increased risk of death for 4DR-PWH [adjusted HR (aHR) 1.68, 95% CI 1.07–2.63, *P* = .024] when compared with non-4DR-PWH. The magnitude of the association was attenuated in the alternative model adjusting for comorbidities and AIDS [aHR 1.57, 95% CI 1.00–2.48, *P* = .052] and lost after adjusting also for baseline CD4 cell count [aHR .93, 95% CI .57–1.53, *P* = .783] (Supplementary Table 2). In the mediation analysis, 4DR status showed a significant indirect effect on mortality via CD4 [aHR 1.62, 95% CI 1.38–1.89, *P* < .001], while the direct effect was not significant [aHR = 1.03, 95% CI .64–1.66, *P* = .915].

**Table 1.** Characteristics of the Study Population

Characteristics	Overall	NO 4DR-PWH	4DR-PWH^a^	*P* Value
	*N* = 1097	*N* = 869	*N* = 228	
Age at BL, y, median (IQR)	51 [46–55]	51 [46–55]	50 [44–55]	.229
Male sex assigned at birth, *n* (%)	809 (73.7%)	644 (74.1%)	165 (72.4%)	.655
Caucasian ethnicity, *n* (%)	1050 (98.1%)	837 (98.8%)	213 (95.5%)	.003
Missing = 27				
Mode of HIV acquisition, *n* (%)				.215
Heterosexual intercourse	235 (21.4%)	178 (20.5%)	57 (25.0%)	
Homosexual/bisexual intercourse	282 (25.7%)	224 (25.8%)	58 (25.4%)	
Intravenous drug use	329 (30.0%)	272 (31.3%)	57 (25.0%)	
Other/unknown	251 (22.9%)	195 (22.4%)	56 (24.6%)	
Years since HIV diagnosis at BL, y, median (IQR)	21.7 (17.4–26.6)	21.8 (17.5–26.7)	21.4 (17.1–26.4)	.641
ART duration at BL, median (IQR)	18.0 (14.6–21.4)	17.9 (14.6–21.4)	18.2 (14.6–21.2)	.883
BL HIV-RNA <50 cp/mL	795 (72.6%)	776 (89.3%)	19 (8.4%)	<.001
Missing = 2				
BL HIV load,^b^ copies/mL, median (IQR)	14.0 (0.90–70.0)	0.90 (0.90–39.0)	1390 (132–19 702)	<.001
Missing = 2				
BL CD4+ T-cell count, cells/mm^3^, median (IQR)	610 (404–830)	661 (466–871)	401 (211–600)	<.001
Missing = 6				
BL CD8+ T-cell count, cells/mm^3^, median (IQR)	928 (651–1255)	896 (651–1230)	988 (648–1386)	.157
Missing = 214				
BL CD4+/CD8 + ratio, median (IQR)	0.64 (0.39–0.97)	0.70 (0.45–1.05)	0.37 (0.21–0.61)	<.001
Missing = 214				
CD4+ T-cell nadir, cells/mm^3^, median (IQR)	188 (73.0–307)	215 (100–329)	92.0 (23.2–186)	<.001
Missing = 14				
Previous AIDS defining event, *n* (%)	277 (25.3%)	199 (22.9%)	78 (34.2%)	<.001
Number of drugs in the ART regimen ongoing at BL, median (IQR)	3 (3–3)	3 (3–3)	3 (2–4)	<.001
Missing = 5				
Regimen composition at BL, *n* (%)				
NRTI	846 (77.5%)	719 (82.8%)	127 (56.7%)	<.001
NNRTI	294 (26.9%)	226 (26.0%)	68 (30.4%)	.224
PI	631 (57.8%)	485 (55.9%)	146 (65.2%)	.015
DRV/r	237 (21.7%)	141 (16.2%)	96 (42.9%)	<.001
DRV/c	36 (3.30%)	20 (2.30%)	16 (7.14%)	.001
ATV/r	135 (12.4%)	133 (15.3%)	2 (0.89%)	<.001
ATV/c	4 (0.37%)	2 (0.23%)	2 (0.89%)	.188
LPV/r	74 (6.78%)	59 (6.80%)	15 (6.70%)	1.000
INSTI	407 (37.3%)	232 (26.7%)	175 (78.1%)	<.001
DTG	129 (11.8%)	90 (10.4%)	39 (17.4%)	.005
RAL	227 (20.8%)	101 (11.6%)	126 (56.2%)	<.001
BIC	19 (1.74%)	18 (2.07%)	1 (0.45%)	.148
EVG/c	30 (2.75%)	21 (2.42%)	9 (4.02%)	.282
MVC	90 (8.2%)	40 (4.6%)	50 (22.3%)	<.001
ENF	15 (1.37%)	2 (0.2%)	13 (5.8%)	<.001
Coinfections diagnosed before BL				
HCV	423 (38.6%)	350 (40.3%)	73 (32.0%)	.028
Chronic HBV	79 (7.2%)	64 (7.4%)	15 (6.6%)	.791
Smoking habits at BL, *n* (%)				.271
Non smoker	387 (35.3%)	299 (34.4%)	88 (38.6%)	
Current smoker	484 (44.1%)	383 (44.1%)	101 (44.3%)	
Former smoker	226 (20.6%)	187 (21.5%)	39 (17.1%)	
Comorbidities diagnosed before BL, *n* (%)				
Diabetes^c^	114 (10.4%)	96 (11.0%)	18 (7.89%)	.205
MACE^d^	57 (5.2%)	52 (6.0%)	5 (2.2%)	.033
Hypertension^e^	270 (24.6%)	223 (25.7%)	47 (20.6%)	.137
Dyslipidemia^f^	762 (69.5%)	609 (70.1%)	153 (67.1%)	.431
CKD^g^ diagnosed before BL	61 (5.6%)	51 (5.9%)	10 (4.4%)	.479
Cancer	59 (5.4%)	43 (4.9%)	16 (7%)	.286

Abbreviations: ATV/c, atazanavir/cobicistat; ATV/r, atazanavir/ritonavir; BL, baseline; BIC, bictegravir; CKD, chronic kidney disease; DRV/r, darunavir/ritonavir; DRV/c, darunavir/cobicistat; DTG, dolutegravir; ENF, enfuvisrtide; EVG/c, elvitegravir/cobicistat; HBsAg, HBV surface antigen; HIV, human immunodeficiency virus; INSTI, integrase strand transfer inhibitor; LPV/r, lopinavir/ritonavir; MVC, maraviroc; NRTI, nucleoside reverse transcriptase inhibitors; NNRTIs, non-nucleoside reverse transcriptase inhibitors; PI, protease inhibitors; DRV/r, darunavir/ritonavir; RAL, raltegravir.

^a^A total of 172 individuals (75.4%) had INSTI-class resistance documented by genotypic resistance test, while 56 (24.6%) met criteria based on virological failure (8 on dolutegravir, 3 on elvitegravir, and 45 on raltegravir).

^b^Negative HIV load was considered as <1 copies/mL, whereas HIV load under the lower detection limit (20 copies/mL) was considered as <20 copies/mL.

^c^MACE defined as cardiovascular death, myocardial infarction, unstable angina, stroke, transient ischemic attack, peripheral arterial ischemia, and coronary, carotid or peripheral artery revascularization.

^d^Diabetes mellitus defined as fasting glucose ≥126 mg/dL at 2 measurements, oral glucose tolerance test 2 h value ≥200 mg/dL, or glycated hemoglobin ≥48 mmol/mol (≥6.5%).

^e^Arterial hypertension defined as blood pressure >140/90 mmHg, confirmed with home blood pressure measurement or 24 h blood pressure monitoring, or use of antihypertensive drugs.

^f^Dyslipidemia defined as total cholesterol ≥200 mg/dL, HDL cholesterol <40 mg/dL, LDL cholesterol ≥130 mg/dL, triglycerides ≥150 mg/dL, or use of hypolipidemic drugs.

^g^CKD defined as estimated glomerular filtration rate <60 mL/min/1.73 m^2^ calculated from the CKD-EPI creatinine equation at 2 measurements >3 m apart.

## DISCUSSION

In our study, 4DR-PWH appeared at increased risk of death compared with non-4DR-PWH. This increased risk appears mainly due to a high prevalence of previous AIDS and a lower CD4 cell count in 4DR-PWH. The advances in ART now enable a substantial proportion of HTE to regain virological control thus improving survival [9]. In our study, fewer than 10% of 4DR-PWH were in a condition of virological control (HIV-RNA <50 copies/mL) at index data, compared with 90% of matched non-4DR-PWH. This significant gap goes in parallel with the risks of adverse clinical outcomes related to persistent inflammation caused by uncontrolled HIV replication, period of treatment interruption and toxicities due to complex ART [4–6]. In our study, one of the matching criteria was the duration of ART at the time 4DR status was reached. Thus, it is expected that 4DR status itself encapsulates the complexity of ART history, including previous treatment failures and AIDS-defining events such as the period spent with a nonsuppressed viremia. This hypothesis is partially supported by our alternative model, in which we adjusted for previous AIDS-defining events and found only a slightly attenuated risk of death in 4DR-PWH compared with non-4DR-PWH. Nevertheless, when CD4 cell count was introduced in the model no evidence of a difference was observed between the 2 groups suggesting that immune-suppression plays an important prognostic role and likely explain the between group differences.

Comparison with other studies is limited by differences in HTE definitions and population heterogeneity. Nevertheless, our findings are in line with the EuroSIDA multicohort in which HTE individuals had a significantly higher crude rate of new AIDS-related clinical events or death compared with controls [11]. This excess risk was largely explained by differences in baseline CD4 counts as confirmed in our mediation analysis [11].

In the OPERA cohort, crude mortality incidence was higher among HTE individuals than non-HTE PWH (2% vs 1% at 2 years, respectively) [12].

The BRIGHTE study reported higher 5-year mortality in its nonrandomized cohort (20%) than in its randomized cohort (6%), where fostemsavir was tested in HTE PWH [13]. Our study's 5-year death probability was 4.62%. Differences in baseline CD4 counts and HIV RNA levels likely explain the BRIGHTE nonrandomized cohort's worse outcomes when compared with our cohort. Comparable mortality between our cohort and BRIGHTE's randomized cohort, where at least 1 or 2 active antiretroviral agents were required, highlights the importance of novel antiretrovirals in achieving viral suppression.

The CAPELLA study reported a 2-year mortality rate of 4.2% (8.3% in the nonrandomized cohort) among HTE PWH receiving lenacapavir. Participants had higher baseline HIV RNA (4.5 log10 copies/mL) and lower median CD4 counts (210 cells/mm³) than our 4DR PWH cohort likely explaining their poorer outcomes [14].

Our study has several limitations. First, the small sample size of 4DR PWH limits statistical power to detect significant differences. Second, the non-4DR PWH group, drawn from a single center, may not fully represent the single study site counterpart of the registry's non-4DR PWH and exposed the study to selection and information bias. Third, the retrospective design may introduce bias due to missing variables. Fourth, up to 30% of 4DR PWH lacked cause-of-death data, complicating comparisons with non-4DR PWH. Fifth, PWH were included from a single country limiting the generalizability to other settings.

In conclusion, 4DR PWH had a higher risk of death than non-4DR PWH, primarily due to lower CD4 cell counts. The priority for this vulnerable population is achieving virological control to enable immune recovery.

## Supplementary Material

ciaf421_Supplementary_Data

## Supplementary Data

Supplementary materials are available at *Clinical Infectious Diseases* online. Consisting of data provided by the authors to benefit the reader, the posted materials are not copyedited and are the sole responsibility of the authors, so questions or comments should be addressed to the corresponding author.
