# Supplementary material for: All-Cause Mortality in People With Four-Class Drug-Resistant HIV: A Matched Cohort Analysis With Data From the PRESTIGIO Registry
Source: Clin Infect Dis. 2025 Aug 1;81(5):e370–4. doi: 10.1093/cid/ciaf421 (PMC12728292; doi:10.1093/cid/ciaf421)
Supplement: ciaf421_Supplementary_Data [file ciaf421_supplementary_data.docx]

Supplementary Figure 1. Kaplan Meier curve of risk of death according to being exposed or not to 4DR.


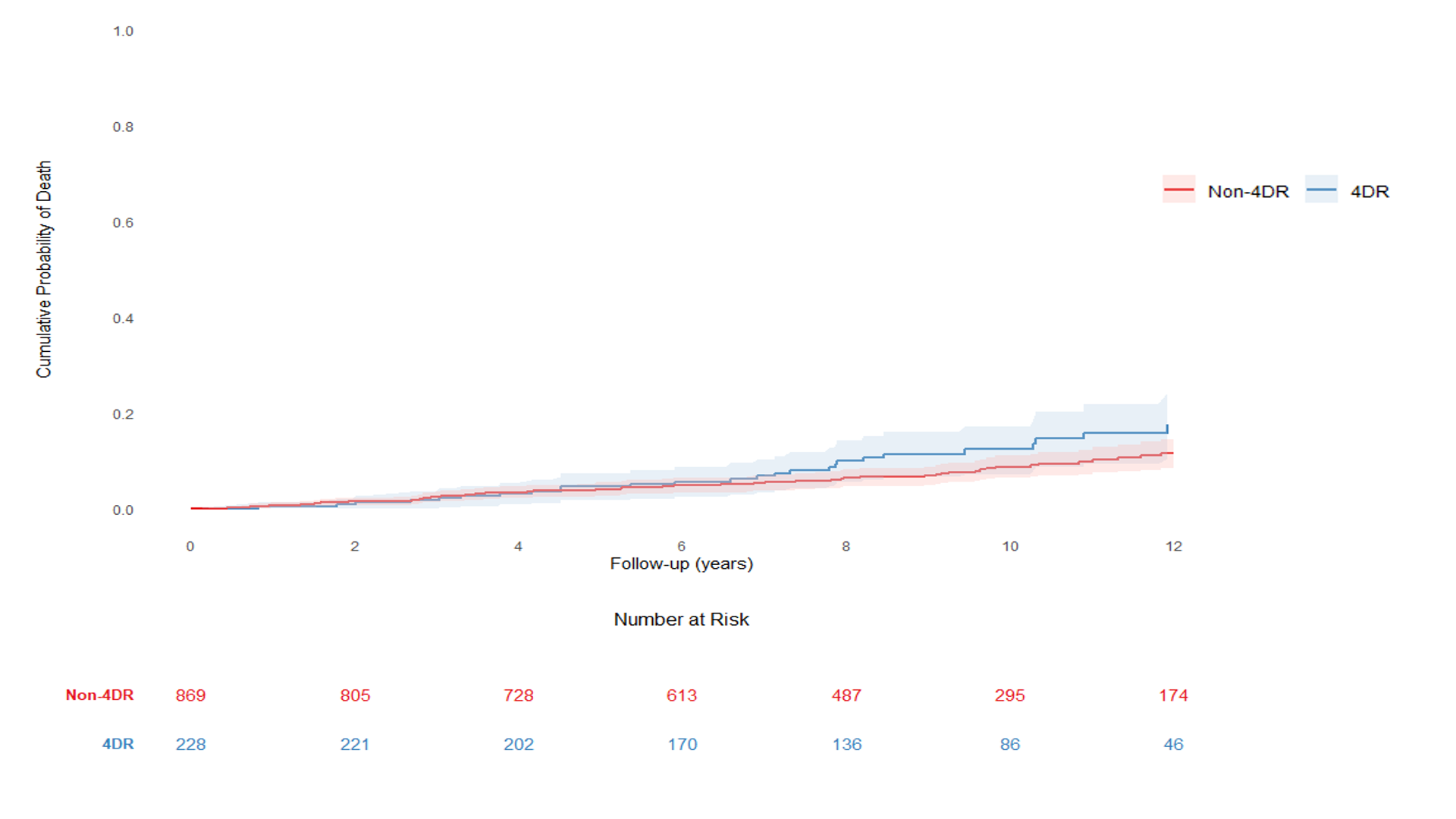


Supplementary Table 1. Causes of death.

| Causes of death, n (%) | Non-4-DR PWH  N=67 | 4-DR PWH  N=28 |
| --- | --- | --- |
| AIDS | 5 (7.5%) | 3 (10.7%) |
| LIVER | 14 (20.9%) | 0 (0%) |
| Non AIDS cancer | 20 (29.9%) | 5 (17.9%) |
| Other non AIDS | 16 (23.9%) | 10 (35.7%) |
| Suicide | 1 (1.5%) | 1 (3.6%) |
| Unknown | 11 (16.4%) | 9 (32.1%) |

Supplementary Table 2. Multivariable Cox models of risk of death.

| Model 1 | aHR | 95% CI | p value |
| --- | --- | --- | --- |
| 4DR vs Non-4DR | 1.70 | 1.08-2.66 | 0.021 |
| MACE^a^ | 1.64 | 0.76-3.56 | 0.209 |
| CKD^b^ | 3.64 | 1.97-6.71 | <0.0001 |
| Diabetes^c^ | 1.69 | 0.98-2.91 | 0.057 |
| HCV | 2.11 | 1.39-3.20 | 0.0004 |
| Neoplasm | 2.33 | 1.22-4.42 | 0.010 |
| Arterial hypertension^d^ | 1.43 | 0.88-2.33 | 0.145 |
| Model 2 | **aHR** | **95% CI** | **p value** |
| 4DR vs Non-4DR | 1.57 | 1.00-2.48 | 0.052 |
| MACE^a^ | 1.62 | 0.74-3.51 | 0.225 |
| CKD^b^ | 3.27 | 1.78-5.99 | 0.0001 |
| Diabetes^c^ | 1.66 | 0.96-2.87 | 0.067 |
| HCV | 2.08 | 1.37-3.17 | 0.001 |
| Neoplasm | 2.20 | 1.16-4.16 | 0.016 |
| Arterial hypertension^d^ | 1.39 | 0.86-2.25 | 0.177 |
| AIDS | 2.11 | 1.40-3.17 | 0.0004 |
| Model 3 | **aHR** | **95% CI** | **p value** |
| 4DR vs Non-4DR | 0.93 | 0.57-1.53 | 0.783 |
| MACE^a^ | 1.56 | 0.72-3.38 | 0.257 |
| CKD^b^ | 2.63 | 1.43-4.83 | 0.002 |
| Diabetes^c^ | 1.72 | 1.00-2.98 | 0.050 |
| HCV | 1.97 | 1.29-3.00 | 0.002 |
| Neoplasm | 1.95 | 1.03-3.69 | 0.041 |
| Arterial hypertension^d^ | 1.52 | 0.94-2.45 | 0.089 |
| AIDS | 1.70 | 1.12-2.58 | 0.013 |
| CD4 cell count. Per 100/mm3 more | 0.80 | 0.73-0.87 | <0.0001 |

List of abbreviations: CI, confidence interval; aHR, adjusted Hazard Ratio; 4DR, four drug class resistence; MACE, major cardiovascular events; CKD, chronic kidney disease.

^a^MACE defined as cardiovascular death, myocardial infarction, unstable angina, stroke, transient ischaemic attack, peripheral arterial ischaemia, and coronary, carotid or peripheral artery revascularization.

^b^CKD defined as estimated glomerular filtration rate <60 mL/min/1.73 m2 calculated from the CKD-EPI creatinine equation at two measurements >3 months apart.

^c^Diabetes mellitus defined as fasting glucose ≥126 mg/dL at two measurements, oral glucose tolerance test 2 h value ≥200 mg/dL or glycated haemoglobin ≥48 mmol/mol (≥6.5%).

^d^Arterial hypertension defined as blood pressure >140/90 mmHg, confirmed with home blood pressure measurement or 24 h blood pressure monitoring, or use of antihypertensive drugs.
